# Supplementary material for: Chromothripsis during telomere crisis is independent of NHEJ, and consistent with a replicative origin
Source: Genome Res. 2019 May;29(5):737–49. doi: 10.1101/gr.240705.118 (PMC6499312; doi:10.1101/gr.240705.118)
Supplement: Supplemental Material [file supp_gr.240705.118_Supplemental_file_1.zip › contigs/annotated_contigs/DB53/contig.3.DB53_length_451_mean_cov_2.26385809313.docx]

**DB53_length_451_mean_cov_2.26385809313**

```
AGGACAA|GATCCAGCTTGAACCTCCAAGTCATTTCACAAGGAAATATGAAATTGCAGAACAGTGTGGGTGGGCAATGGACATGACCTG
        >chr8:75618381-75618499 + E=2e-59 p=5e-02
ACTTTCATTTATTAAATAAACACAGACTTGAGT|AGTT|TTGTCCCTTCATAAATCTCCATGAGCAACCCCCTCTAAGTCATCCCTCAT
                                  >chr8:75618556-75618839 + E=7e-159
AAATTTACAATCTAAGTAATCTAAGTAAAATCAAGTGTGCTTGCTCTGTAATGTAGCTTCCAGGCAAATTAATTAGCGTTCACTTCCAA

AAACCTTCTCACTCCTAAGTCCTTTCTATAGCGAAATTCAGGAGCTGCCAGTGTTAGTCCAACTCCCAAGATTATGTGCAATGTTGGTT

CTTGCACGAACCAGAGCACGAGGGGCCTCCTTGGTCTCCAGTTCTC|TTGTA|CTTCTGAGAACATTAGCAGTGGCATTGCCAAGGTAT
                                               >chr8:75618497-75618549 + E=1e-19
AGCACTTGGGG
```
